# The Prevention of Shock

**Published:** 1899-03-18

**Authors:** 


### THE PREVENTION OF SHOCK.

Writing on the subject of shock, Dr. Robert Dawburn,[1] of New York, advises that in cases of operation where the advent of shock seems a probability free use should be made of hot intravenous saline infusion, injected while the patient is still upon the operating-table and asleep from the anæsthetic. When speed is not important and a prompter stimulant effect is not essential the rectal route is an excellent one. Even during an operation the maintenance of a full large bowel by means of a rectal irrigator can only be of benefit, and is an additional safeguard against dangerous chloroform narcosis by maintaining filled vessels. In bad cases it may be well to keep up this "hot colonic irrigation" alternately, an hour on and an hour off, for, perhaps, half a day. "When following confinement the accoucheur has a partly exsanguined patient, he adds wonderfully to her comfort if he injects high up a couple of litres of very hot salt water by rectum. . . . Not a drop of the amount thus poured into the colon will ever be seen again." For intravenous injection the fluid should be a saline solution formed by adding six parts of common table salt to a thousand of water, boiled and filtered; roughly, a heaped-up teaspoonful to the quart. It is most important to remember that plain warm water devoid of sodium chloride should not be used intravascularly. "It is certain that whosoever tries this will, if the water be used in the customary large amount, kill his patient with quite indecorous speed by a wholesale disintegration of the red discs." Dr. Dawburn insists upon the temperature of the fluid injected being higher than the normal temperature of the blood. "The proper temperature for the injection fluid is as hot as the hand can bear, and this is about 120 deg. Fahr., or 49 deg. C. (Of course, the temperature at the heart, when the great bulk of blood has diluted the slowly entering fluid, will be much lower than this.) There need be no fear of injuring the blood or other tissue by such heat. Very many times the writer has now used it, and never with cause for subsequent regret. This point seems not at all well known as yet in the profession, and nearly all the text books still advise infusion at about 100 deg. The higher temperature here recommended is very stimulating to the flagging heart." As to the amount of fluid to be used this may be set down as never less than a litre (a pint and three-quarters), often two litres, and occasionally even three. Small quantities are of no use. The kidneys readily remove any excess, but where these are diseased a less amount of water than usual must be employed. It is important to inject slowly into the vein. The time occupied should never be less than ten minutes per litre. If the injection has done good, but the effect seems to be passing off, the rectal hot douche may be useful, the foot of the bed all the time being raised. In regard to the treatment of shock when once developed, the practitioner is strongly urged not to attempt to feed or give medicine by the mouth for fear of producing nausea. This is itself but one type of shock, and no one should take the least risk of adding it to the already existing surgical shock. The duration of shock is not long, and the patient may easily have his strength sustained during that time without using his stomach.

[1] Med. News, Feb. 25.

